# Microbiome dysbiosis in lung cancer: from composition to therapy

**DOI:** 10.1038/s41698-020-00138-z

**Published:** 2020-12-10

**Authors:** Ning-Ning Liu, Qiang Ma, Yang Ge, Cheng-Xiang Yi, Lu-Qi Wei, Jing-Cong Tan, Qiao Chu, Jing-Quan Li, Peng Zhang, Hui Wang

**Affiliations:** 1 State Key Laboratory of Oncogenes and Related Genes, Center for Single-Cell Omics, School of Public Health, Shanghai Jiao Tong University School of Medicine, 200025 Shanghai, China; 2 Department of Thoracic Surgery, Shanghai Pulmonary Hospital Tongji University, Shanghai, China

**Keywords:** Lung cancer, Outcomes research, Infection, Lung cancer, Cancer microenvironment

## Abstract

The correlations between microbiota dysbiosis and cancer have gained extensive attention and been widely explored. As a leading cancer diagnosis worldwide, lung cancer poses a great threat to human health. The healthy human lungs are consistently exposed to external environment and harbor a specific pattern of microbiota, sharing many key pathological and physiological characteristics with the intestinal tract. Although previous findings uncovered the critical roles of microbiota in tumorigenesis and response to anticancer therapy, most of them were focused on the intestinal microbiota rather than lung microbiota. Notably, the considerable functions of microbiota in maintaining lung homeostasis should not be neglected as the microbiome dysbiosis may promote tumor development and progression through production of cytokines and toxins and multiple other pathways. Despite the fact that increasing studies have revealed the effect of microbiome on the induction of lung cancer and different disease status, the underlying mechanisms and potential therapeutic strategies remained unclear. Herein, we summarized the recent progresses about microbiome in lung cancer and further discussed the role of microbial communities in promoting lung cancer progression and the current status of therapeutic approaches targeting microbiome to alleviate and even cure lung cancer.

## Introduction

The human body coexists with a complex array of commensal microbiome that colonizes the host microenvironment forming a dynamic micro-ecological system developed during evolution. The commensal microbiome shares a symbiotic relationship with the host during the long-term coexistence development which eventually forms a dynamic microecosystem^1^. Currently the human microbiome has received extensive attention and been demonstrated to play a critical role in various aspects of human health and disease status via immunity, metabolism and inflammation^2^.

Lung cancer poses a great threat to global public health and is ranked as the most common cancer (11.6% of all cancers) with over 2.09 million diagnosis and 1.7 million deaths worldwide in 2018^3,4^. It is generally divided into two histological-pathological types including non-small cell lung cancer (NSCLC) and small cell lung cancer (SCLC). Treatments for lung cancer include surgery, chemotherapy, radiotherapy, targeted therapy and emerging immunotherapy. And the best recommendations depend on patient’s TNM stage and unique health situations^5^. However, most patients are diagnosed at an advanced stage, with high mortality and poor benefit from limited treatment options^6^. On the other hand, multiorgan metastatic and relapse in pre-treatment and post-treatment are critical causes of death without effective therapy. There are growing emergency and social demand in exploring the carcinogenesis and new therapeutics for this deadly disease. Lung cancer has been widely considered to be a complicated disease caused by interactions between host and environmental factors^7^. Among diverse environmental risk factors, microbes present a vital part in maintaining microecological balance and regulating host immune responses to multi-treatments. Although the healthy lung tissues were long considered as a sterile environment, it was found recently that there were certain microbial species existed in the lung tissues impacting the balance between health and pathogenesis in the lung microenvironment with the advancements of high-throughput next generation sequencing (NGS) technologies^8,9^. Increasing studies have profiled the microbiome in respiratory samples from healthy adult lungs^10,11^. It has discovered the most common phyla including *Bacteroides*, *Firmicutes*, and *Proteobacteria* and genera like *Streptococcus*, *Pseudomonas*, *Veillonella*, and *Prevotella*^12^. Nowadays researches in the lung microbiome and important discoveries in the microbiome’s association with lung diseases are growing rapidly. It is believed that improved understanding of this association will provide novel insights into the pathogenesis of lung diseases. In this review, we summarized and evaluated the current development of the interplay and the underlying mechanisms between microbiota and lung cancer. Furthermore, we also discussed the prospects about the carcinogenesis and therapeutic applications of microbiome on lung cancer.

## Lung microbiome and gut microbiome

Microbiome was defined by the bacteria, fungi, virus, protozoa and their related genes and genomes, as well as metabolites^13^. At present, more and more efforts have been focused on the most compelling commensal microbial ecosystem interacting with the human body—especially the gut microbiome which is considered as a forgotten organ mediating host homeostasis via complex mechanisms^14^. Emerging gut microbiota research are fully facilitated by the rapid development and application of high-throughput molecular technologies^15^, bioinformatics and metagenomics^16,17^. It was reported that gut microbiome was closely correlated with various chronic diseases such as gastroenteric cancers within which carcinogenic *H. pylori* was colonized frequently, as well as multiple pathological processes of remote organs in the host^18,19^. Conversely, lungs were regarded as a pair of sterile organs until new studies assisted with high-throughput sequencing technologies challenged the old false dogma^20^. It is now generally recognized that microbial disturbances influence a variety of lung diseases. The lung microbiota is composed of bacteria, fungi, and virus, which derived from the inhalation of mucosal secretions, nasopharynx, oropharynx, and environmental air exchange^8,21–23^. Under healthy conditions, bacteria of the genus *Propionibacterium, Streptococcus, Haemophilus*, and *Veillonella* coexist with fungi such as *Aspergillus*, *Penicillium*, and *Candida*, but they do not cause infection of human lungs^24^. It is not surprising when considering multiple unknown interactions in other tissues. All these interactions among microbiome, immunity and metabolism in these microbial niches affect multiple lung pathogenesis of COPD, asthma, cystic fibrosis and lung cancer^9,12,25–28^. Recently, increasing interests have been raised about the field including commensal lung microbiome communities and the possible mechanisms maintaining microecological effects on human respiratory system. Based on previous investigations, several controversial hypotheses have been proposed including “Microbiota-Brain-Gut axis”^13^, “Microbiota-Gut-Liver axis”^29^, and “Microbiota-Gut-Skin axis”^30^.

**Fig. 1:**
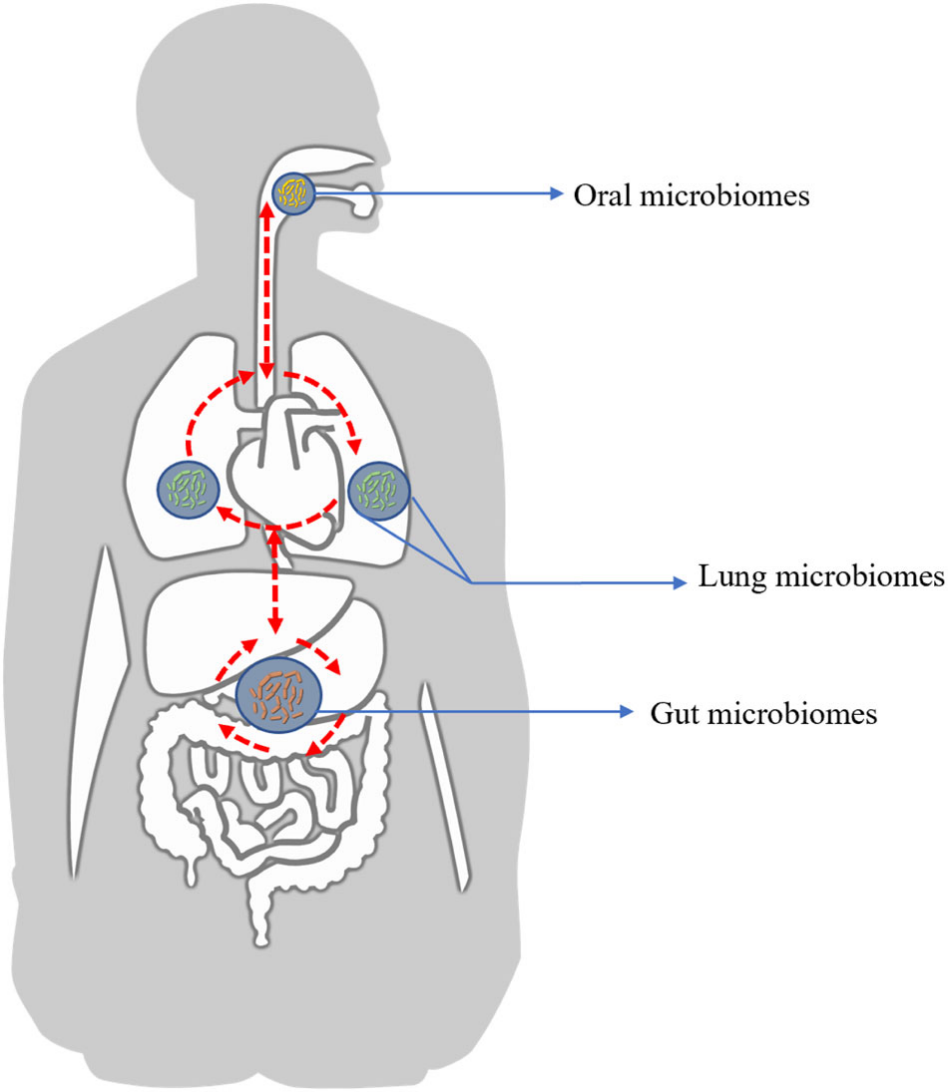
Dynamic connections of microbiome inhabiting different human body sites. Oral, lung, and gut microbiome could communicate with each other via direct manner including mucosal dispersion, respiratory and digestive activities, and indirect manner via inflammatory substances, cytokine, and metabolites in systematic circulation. Bacteria and its metabolites from intestinal tract modulate the differentiation tendency of naive T cells and Th17 release, modulating the systematic inflammation and immunity.

In fact, the human body is a dynamically balanced integrity and microorganisms in various body sites can interact with each other directly including mucosal dispersion, respiratory and digestive activities, or indirectly via inflammatory substances, cytokine, and metabolites in systematic circulation as shown in Fig. 1, which exhibited the possible microbial communication between the oral cavities and lungs, as well as gut. The alterations of local lung microbiome communities mainly depend on three aspects which can be summarized as microbial migration, elimination and growth rates under the condition of health and disease^8,9,31^. Some researchers have brought forward that oral microbiome may be the primary sources of lung microbiome from a long-term and validated observation (through swallowing, mucosal dispersion, and micro-aerosols or secretions generated in oral cavities)^31^. The respiratory tract and gut could communicate with each other via biological processes including micro-aspiration and inhalation^32^. Bacterial metabolites from the intestinal tract can regulate the differentiation tendency of naive T cells, effector T cells, Tregs, or Th17 release, which further induces systematic inflammation and immunity response^33^. Furthermore, they could be transported into host bloodstream to regulate the systemic immune activity and alter the microbial communities located in respiratory tract^25,34,35^. Although some theoretical models had been put forward, no specific studies were conducted to approach the relationship and communicating manners in terms of the species diversity and the relative abundance of microbiota at different sites within respiratory and gastrointestinal tract.

The temperature and pH environment in gastrointestinal tract are relatively constant and migration of microbes is unidirectional and always altered by complicated physical and chemical conditions. By contrast, the lung is frequently exchanging gas with outside environment to maintain abundant reserves of oxygen and microbiota. Furthermore, there is no physical barrier and gradient diversity of pressure and temperature in upper respiratory tract which provide bidirectional conditions for lung-resident microbial migration and dynamic changes^36^. On the other hand, Lozupone et al. reported that the progressive maturation pattern of diversity, stability, and resilience of the lung microbiota from birth to adult mice was consistent with both human lung and gut microbiota during the first 3 years^37^. Previous studies revealed that early-life formation of microbiota and immunologic environment in human airways and gastrointestinal tract may be derived from the skin and external environment^38–40^. Despite distinct differences in micro-anatomic features, composition and population dynamics in gut and lung microbiota, these two organs share a similar homeostasis and certain physiological characteristics such as microbiota maturation process, mucosal immune system, co-evolution and communication with immune cells and continuous exposure to outside environment.

Intriguingly, increasing clinical studies indicated that multiple lung diseases were more likely to develop in patients with gastrointestinal disorders^41,42^. These significant discoveries lead us to reconsider whether the microbiome interaction network really exists and modulates host susceptibility to either internal or external pathogenic factors. Gut microbiome have been confirmed contributing to the chronic obstructive pulmonary diseases^43^, the progression of asthma^44^, and the worsening acute lung injury^45^. More and more studies have also implicated the connections and modulatory effects of specific microbial metabolites in gut and lung via circulation^46,47^. For example, a significant reduction of the microbial metabolites including the fatty acids, acetate, butyrate and propionate, as well as isoacids in the feces from patients with bronchial asthma was observed when compared with healthy controls^48^. And *Faecalibacterium prausnitzii* and *Akkermansia muciniphila* have been reported to suppress the inflammatory responses in pediatric allergic asthma through inducing anti-inflammatory cytokine IL-10 and inhibiting the secretion of pro-inflammatory cytokines like IL-12^47^. Previous studies also found that alterations in colonic luminal, serum, and hippocampal metabolomic profiles in mice treated with ketogenic diet were significantly correlated with seizure protection, supporting the distant modulation of intestinal microbiome in other organs^49^. Bingula et al. reported that the gut bacteria and their fragments were taken up into DCs (dendritic cells) and macrophages through phagocytosis and then migrate into gut or lungs to regulate immune response^50^. Tsay et al. demonstrated that gut flora can induce lung inflammatory reaction against bacterial pneumonia and enhance neutrophils infiltration through TLR4 in mice^51^. Taken together, the complex and interventional ecosystems regulate various pathological processes and maintain the physiological equilibrium of gut and lung. Thus, a new hypothesis was put forward as “Microbiota-Gut-Lung axis” based on the diverse and complex gut-lung microbiome networks established based on numerous long-tern epidemiological observations. However, the mechanisms underlie the “Microbiota-Gut-Lung axis” remains elusive and more robust evidences are still required to kindle the lamp.

## Lung microbiome and host metabolism

Dysregulation of host metabolism by microbiome alterations have been intensively studied in the intestinal tract^52–54^. The carcinogen acetaldehyde and deoxycholic acid produced by microbiome were reported to be involved in esophagus and liver carcinogenesis^55,56^. Visconti et al. reported that the blood and fecal metabolites were analyzed together with the characterization of gut microbiome by metagenomic shotgun sequencing. Their findings supported a correlation between active pathways of gut bacteria and metabolites found in feces and blood^57^. Metabolism is essential for maintaining human body homeostasis through numerous pathologic and physiologic processes^58^. There are also emerging studies approaching lung microbiome associated with host metabolism. Cribbs et al. discovered that specific metabolic profiles correlated with bacterial organisms were associated with glycerophospholipid and lineolate pathways, which play an important role in the pathogenesis of pneumonia in HIV-infected individuals through 16s rRNA sequencing in the bronchoalveolar lavage fluid^59^. Bei et al. also found that primary metabolites secreted by *Pseudomonas aeruginosa* using substrates produced by *Rothia mucilaginosa* might contribute to its pathogenesis in the progression of cystic fibrosis^60^. One of the high-profile metabolites is short-chain fatty acids (SCFA) which is produced by large amounts of commensal microbes and acts as a crucial signaling molecule in host cells. And many studies have focused on the role of SCFA in host gut and immunity, while the functions of SCFA in respiratory system, epithelium and immunity remain unclear. It has been shown by Gauguet et al. that mice lacking SCFA in the gut are prone to suffer from more bacterial load like *Staphylococcus aureus* which could be modulated by pulmonary Th17 immunity^61^. Previous study by Cait et al. demonstrated that dietary supplementation with short-chain fatty acids (SCFAs) can ameliorate this enhanced asthma susceptibility by modulating the activity of T cells and DCs in mice^62^. Besides, some researches have remonstrated that modulation of gut microbiome in preclinical model can alter host immune response and susceptibility to pulmonary infection factors^63,64^. Furthermore, SCFAs has been reported to regulate differentiation of bone marrow cell and maintain host immunological homeostasis^65,66^. Under certain circumstances, SCFAs can modulate the composition of gut microbiome and induce myelopoiesis resulting in an anti-inflammatory milieu in the airways^67,68^. Therefore, the concept of “gut-bone marrow-lung axis” has been brought forward, which represents a mechanistic explanation on how the gut microbiota derived SCFAs modulate the host immune system against exogenous pathogenic factors. However, currently most of the studies are focused more on the possible association between the intestinal-pulmonary axis and lung inflammatory diseases with few studies involving lung cancer. Although recent investigations in microbial metabolism associated with cancer occurrence and progression are shedding novel insight, limited evidences are available to establish solid connections concerning distal metabolic regulations between gut and lung and the mechanism still awaits further investigation.

## Lung microbiome and host immunity

The microbiome regulates host immune activity directly or indirectly by mediating host susceptibility to various pathogenic factors and therapeutic outcomes. The dynamic interaction between microbiome and immune system enables host to recognize and prevent bacterial or fungal invasions and infections. In preclinical studies germ-free (GF) mice lacking intestinal microbiome displayed severe immune dysplasia with incomplete mucous layer, immunoglobulin secretion disorder, and decreased size and number of lymph nodes^69^. The special subgroup CD4 + Th17 cells play an important role in microbial interactions, mucosal immunity functions and host response to inflammatory diseases of intestinal tract, lungs and skin^70^. Mantis et al. reported that IgA mainly regulated bacterial virulence in the gut by blocking bacterial adherence to mucosal epithelial cells^71^. The microecology with low-density and less-stable microbiome was susceptible to lose balance of commensal bacteria and be invaded by exogenous pathogens^72^. A recent study revealed that high density of commensal microbiota might promote the clinical outcome of vaccine for infants^73^. Gut microbiota can stimulate Th17 response and modulate the generation of IL-17, which is involved in the elimination of certain pathogens^74,75^. In addition, IL-17 pathway is also involved in the pathogenesis of several pulmonary pathologies including asthma, sarcoidosis, obliterative bronchiolitis, and bone marrow transplant–related pneumonitis^76–79^. Gollwitzer et al. reported that bacteria resident in the lung regulated the expression of certain innate immunity genes including IL-5, IL-10, and IFN, and the expression level of PD-L1 on CD11bC DCs and FoxP3^+^CD25^+^ Treg cells were higher in the lungs of SPF (specific pathogen-free) neonates than GF (germ free) mice^80^. Steed et al. reported that a microbially associated metabolite, desaminotyrosine (DAT), protects host from influenza via enhancing type I IFN stimulation and reducing lung cancer immunopathology^81^. Similarly, Takeshi Ichinohe et al. found that commensal microbiota could regulate immunity in respiratory mucosa through inflammasomes and provided immune activation signals at steady state after influenza virus infection^63^. The most recent study revealed that the fermentable fiber inulin could alter gut microbiota structure and the associated metabolites like short-chain fatty acids which eventually improve the response of mice to influenza virus infection by dampening damage induced by neutrophils and enhancing anti-viral CD8 + T cell responses^67^. And enrichment of lung microbiome with oral taxa was found to be associated with Th17 inflammation, in which TLR4 responses were impacted by lung microbiome composition^25^. Moreover, commensal microbiota was shown to drive proliferation and activation of Vg6 + Vd1 + T cells in lung cancer^82^. Nevertheless, there is no consistent definition of a healthy or beneficial lung microbiota, partly due to limited understanding in approaching the association between lung-resident microbiome and host immunity.

## Contribution of microbiome to cancer

Cancer is generally thought to be a multifactorial pathological process, where normal cells begin to proliferate in an unprogrammed manner resulting in inhibition of apoptosis, autophagy, inflammations and DNA damage. There are increasing commensal and pathogenic microorganisms defined in the human body with reported carcinogenic properties and most of them are significantly correlated with carcinogenesis epidemiologically^83^. Here, we made a summary of previous studies in microbiome correlated with lung cancers have been listed in the Table 1^10–12,82,84–94^. The results demonstrated the close relationship between microbial communities and respiratory tract. The initiations of surface boundary tumors are often associated with the host mucosal immune barrier destruction. When the mucosal surface is damaged, the microenvironment of the original tissue and commensal microbiome will be reconstructed if the injury cannot be repaired in time. Otherwise, this damage will continue to intensify and lead to recurring inflammation which may induce cancer at the end. It is possible that microbiome located in the surface-bound tumor or intratumor utilize tumor-derived carbon sources and other nutrients to interact with the tumor immune microenvironment in the long-term coexistence^95,96^. Of note, the most significant microbial carcinogenetic effect is the chronic infections by HBV and HCV induced liver cancer development^97^. Interestingly, a recent study found that the fungal microbiome was abundantly enriched in pancreas and triggered pancreatic carcinogenesis via MBL activation^98^. In aggregate, increasing evidences have supported that dysbiosis of commensal microbial communities can alter the host susceptibility to carcinogenic factors. These findings reflecting the carcinogenesis development can provide a novel insight to help us understand the process from normal tissue to precancerous lesions and to advanced lung cancer.

**Table 1:** Summary of lung cancer microbiome.

Categories	Phylum/Genus	Sample source	Major findings
Bacteria	*Staphylococcus, Streptococcus, Lactobacillus, Pasteurellaceae, Herbaspirillum, Sphingomonadaceae, Aggregatibacter*, and *Lactobacillus* etc	Paired mouse lung cancer and normal tissues	Commensal microbiota induced γδ T cells promote inflammation and lung cancer development^82^
	*Capnocytophaga* and *Veillonella*	Human saliva	*Capnocytophaga* and *Veillonella* were significantly enriched in the saliva from lung cancer patients^92^
	*Streptococcus, Veillonella* (smaple testing)*; Veillonella, Prevotella*, and *Streptococcus* (in vitro)	Human air brushes	The enrichment of the lower airway microbiota with oral commensals was relevant to the upregulation of lung cancer pathogenesis ERK and PI3K signaling pathways^91^
	*Veillonell, Megasphaera, Actinomyces, Arthrobacter, Capnocytophaga, Rothia, Streptococcus*, and *Veillonella*	Human BALF	In different metastatic states of lung cancer, differential genera between squamous cell carcinoma and adenocarcinoma were different. And in different histologic types of lung cancer, distant metastasis-related genera were not the same^12^
	*Bifidobacterium, Aecalibacterium, Bacillus, Streptococcus infantis, Veillonella* etc	Human feces	13 selected gut microbial signatures can be established for the potential prediction of the ref. ^94^
	*G. adiacens, Enterococcussp, Streptococcu intermedius, Escherichia coli, Streptococcus viridans, Acinetobacter junii, and Streptococcus sp*.	Human sputum	*G. adiacens* and associated correlated microbes were significantly correlated with lung cancer status and stage^10^
	*Proteobacteria*	Human BALF	A predominance of proteobacteria existed both in cancerous lungs and other airway disorders^11^
	*Acidovorax*	Paired human lung cancer and tumor tissues	Mutations in TP53 were correlated with the presence of *Acidovorax* in the lung microenvironment^84^
	*Proteobacteria, Firmicutes*, and *Bacteroidetes*	Paired human lung cancer and tumor tissues	A significantly lower abundance of *Proteobacteria (Acinetobacter and Acidovorax*) and higher prevalence of *Firmicutes (Streptococcus)* and *Bacteroidetes (Prevotella)* in lung cancer patients compared to emphysema-only patients^89^
	*Thermus* and *Legionella*	Paired human lung cancer and tumor tissues	*Thermus* is more abundant in tissue from advanced stage patients and *Legionella* is higher in patients who develop metastases^93^
	*Veillonella* and *Megasphaera*	Human BALF	*Veillonella* and *Megasphaera* were relatively more abundant in lung cancer patients^86^
	*Granulicatella, Abiotrophia* and *Streptococcus*	Human oral and sputum samples	*Granulicatella, Abiotrophia*, and *Streptococcus* were significantly enriched in lung cancer patients attributed to household coal burning exposures compared to healthy controls^85^
	*Streptococcus* and *Neisseria*	Paired human lung cancer and tumor tissues	The abundance of genus *Streptococcus* and *Neisseria* displayed an increasing trend from healthy to noncancerous to cancerous site^88^
	Bacteroidaceae, Lachnospiraceae, and Ruminococcaceae	Paired human lung cancer and tumor tissues	Greater abundance of Bacteroidaceae, Lachnospiraceae, and Ruminococcaceae were associated with reduced RFS or DFS^90^
Virus	HPV	NHIRD	There was a significant increase in lung cancer risk among Taiwanese women who were exposed to HPV infection^87^

*BALF* bronchoalveolar lavage fluid, *RFS* recurrence free survival, *DFS* disease free survival, *HPV* (human papillomavirus, *NHIRD* (National Health Insurance Research Database.

Genotoxic toxins or metabolites produced by bacteria can directly damage host DNA and induce genomic instability via reactive oxygen or nitrogen species or natural killer immune receptors which cause cancer-like characteristic alterations when cumulative damage effect overrides the host self-repair ability^99^. Until now, more and more attention has been paid to intestinal microbiota associated with colorectal cancer development, which has also provided us a better understanding about contributions of microbiome to cancer^100^. A most recent study by Manzano et al. revealed a distinct mutation signature from organoids injected with genotoxic pks+ *E. coli*, which indicated a direct role of bacteria in the occurrence of oncogenic mutations^101^. Multiple signaling pathways are involved in the process of carcinogenesis associated with microbiome interventions^102–104^. In addition, destruction of boundary surface between host and microbes could also activate pattern recognition receptors and their signaling cascades which leads to imbalance in the symbiotic microenvironment. The existence of gut microbes was also involved in other aspects of cancer initiation and progression such as angiogenesis, invasion, tumor immune microenvironment and apoptosis which can be promising research hotspots for in-depth investigation^105^.

Prior studies have uncovered some correlations between microbiome and lung inflammatory and organizational structure in several respiratory diseases including COPD (chronic pulmonary disease), IPF (idiopathic fibrosis), asthma, CF (cystic fibrosis) and non-CF Bronchiectasis, as shown in the Table 2^23,26,27,106–125^. Some researchers proposed that a disordered microbial dysbiosis might provoke dysregulation in host physiology and contribute to exacerbations in chronic lung diseases. Several observations reported that respiratory viruses were identified in the respiratory specimens of 39–56% of chronic obstructive pulmonary disease (COPD) patients compared to 6–19% at clinical baseline^126,127^. It was also revealed that pathogenic bacteria existed in 51–70% of patients during disease exacerbations compared to 25–48% in the initial stable clinical baseline^128^. Another large cohort survey reported that CXCL8/IL-8 was significantly associated with lung microbiome diversity and community structure, which can mediate host inflammatory responses during COPD exacerbations in some subjects^129^. Another slowly progressive lung disease is idiopathic fibrosis (IPF) has been confirmed to harbor a distinct microbiome from that of healthy lung status^130^, and a randomized trial reported that antibiotic therapy could be beneficial to survival of IPF patients^131^. Robert et al. revealed that radiographic honeycombing altered the lung microbiota of patients with IPF, which might further exacerbate the anatomic disruption of IPF in a bidirectional interaction^125^. Despite growing evidence of the associations, the causal significance of an altered lung microbiota in COPD and IPF remains elusive. Furthermore, lung microbiome including bacteria or virus infection could potentially invade epithelial cells of the airways inducing host immune response or triggering the wound healing cascade in chronic pathogenic stimuli^132^. Overall, these hypothesis driven theories or epidemiological observations or cohort studies, especially whether the microbiome correlated with chronic lung diseases participate in the exacerbation or initiation of lung cancer, still awaits further investigations. Emerging studies have also raised interests about correlations between lung cancer and microbiome by high-throughput sequencing and epidemiological analysis. It was found that significantly high abundance of *Granulicatella, Thermus, Legionella*, and *Streptococcus* were observed in lung tumor tissues compared with control groups^85,88,93^. Of note, Tsay et al. demonstrated that the enrichment of the lower airway microbiota with oral taxa (*Streptococcus and Veillonella*) was significantly correlated with the upregulation of lung carcinogenic ERK and PI3K signaling pathways. In vitro exposure of airway epithelial cells to *Veillonella, Prevotella*, and *Streptococcus* also led to upregulation of these same signaling pathways^91^. Gomes et al. reported that lung cancer microbiota was enriched in Proteobacteria and more diverse in squamous carcinoma than adenocarcinoma, particularly in males and heavier smokers^11^. Another study by Greathouse et al. revealed that mutations in TP53 were correlated with the presence of *Acidovorax* in the lung microenvironment^84^. The emerging technologies revealed that the niche effects of lung-resident microbiome on lung cancer should not be neglected. From a global perspective, *Pseudomonas, Streptococcus, Staphylococus, Veillonella*, and *Moraxella* were frequently reported as the most relevant lung cancer-related microbiome^10–12,82,84–94,133^. Notably, some lung commensal microbiota including lung cancer-related microbiome including *Proteobacteria, Streptococcus, Bacteroidetes, Veillonella*, and *Moraxella* have also been identified significantly correlated with lung inflammatory diseases^11,26,89,91,92,109,110,115,125^. Yet, limited by technology development and ethics, the nasal secretions, oral saliva, sputum, and BAL fluid are often used indirectly for lung microbiome research. Most studies based on indirect samples and evidences were problematic and failed to illustrate the molecular mechanisms in this field. Despite the inevitable limitations in preclinical animal model and cell lines, emerging advances in organoid technology has allowed for innovative and meaningful investigations of 3D human lung tissues. There are increasing discussions about the feasibility of utilizing lung organoids to approach lung tissue cell–cell interaction mechanism and the potentiality of IL-17 signaling pathway during lung infection^134,135^. Future research should try to use organoids to better simulate and explore the roles of microorganisms in lung cancer and the possible molecular mechanisms due to its success in investigation of microbiota associated with colorectal cancer^101^.

**Table 2:** Possible correlations of lung cancer microbiome with other respiratory illnesses.

Categories	Phylum/Genus	Respiratory illnesses	Major findings
Bacteria	*Streptococcus pneumoniae, Haemophilu influenza, Moraxella Catarrhali*, and *Pseudomonas aeruginosa*	COPD	These bacteria are more colonized in COPD patients epidemiologically, *Pseudomonas aeruginosa* in COPD patients may indicate worse status^107^
	*Proteobacteria, Actinobacteria*	COPD	*Proteobacteria* and *Actinobacteria* may induce a more intense inflammation in severe COPD^26^
	*Proteobacteria* (*particularly Haemophilus spp*) and *Bacteroidetes* (*particularly Prevotella spp*)	COPD	More Pathogenic *Proteobacteria* (particularly *Haemophilus spp*) and less *Bacteroidetes* (particularly *Prevotella spp*) were detected in COPD patients compared to general people^109^
	*Veillonella* and *Prevotella*	COPD	A significant correlation with *Veillonella* and *Prevotella* in BAL in the early COPD patients was identified^23^
	*Gemella*, and *Porphyromonas* etc.	IPF	Radiographic honeycombing can alter lung microbiota of patients with IPF, which may exacerbate the anatomic disruption of IPF in a bidirectional interaction^125^
	*Staphylococcus aureus*	IPF	*Staphylococcus aureus* was frequently observed culture-positivity in the BAL fluid of patients with IPF^113^
	*Staphylococcus sp*. and *Streptococcus sp*.	IPF	*Staphylococcus sp*. and *Streptococcus sp*. were positively correlated with IPS progression and co-trimoxazole with antibiotic therapy can improve condition^108,116^
	*Campylobacter, Stenotrophomonas* and *Veillonella*	IPF	There were increased *Campylobacter* and *Stenotrophomonas* and decreased *Veillonella* in acute exacerbation of IPF compared to stable IPF^111^
	*Streptococcus pneumoniae*	IPF	Streptococcus pneumoniae triggers progression of pulmonary fibrosis through pneumolysin^110^
	*Moraxella*, and *Corynebacterium*	Asthma	Specific bacterial genera are shared between the nasal and the bronchial mucosa which are associated with markers of systemic and bronchial inflammation^27^
	Gram-negative bacteria	Asthma	A component of Gram-negative bacteria, LPS, can decrease asthma level in mice via induction of the ubiquitin-modifying enzyme A20^115^
	*Pseudomonas aeruginosa*	CF	The oral dominant and pathogen (*Pseudomonas Aeruginosa*) can contribute to inflammation and lung structure changes^112^
	*Streptococcus milleri group (SMG)*	CF	*Streptococcus milleri group (SMG)* established chronic pulmonary infections in 39% of acute pulmonary exacerbations^117^
	*Stenotrophomonas maltophilia* or *P aeruginosa*	Non-CF Bronchiectasis	Host genotype (fucosyltransferase 2 secretors) is linked to increased P aeruginosa, which is consistently associated with exacerbations and poorer lung function, clinical outcomes, and mortality^106,118^
	*Proteobacterisa (*e.g., *Haemophilus sp., Pseudomonas sp.)*	Non-CF Bronchiectasis	*Proteobacterisa* occupied major part in microbiome communities in sputum samples from baseline to exacerbation of Non-CF Bronchiectasis^114^
Fungus	*Candia*, *Phialosimplex*, *Aspergillus*, *Penicillium*, *Cladosporium*, and *Eutypella*	COPD	COPD patients have personalized structures and varieties in sputum microbial community during hospitalization periods^123^
	*Aspergillus*	COPD	*A. fumigatus* senitization is related to poor lung function and positive filamentous fungal culture is a common feature of COPD^119^
	*Aspergillus*	IPF	Infection with aspergillosis contributes to chronic fibrosing pulmonary aspergillosis, which may result in chronic scarring of the lungs^120–122^
	*Alternaria alternata* and *Cladosporium herbarum*	Asthma	A large cross-sectional study of 1132 adults with asthma found that senitization to *Alternaria alternata* or *Cladosporium herbarum* is a significant risk factor for severe asthma in several European countries and Australia, New Zealand, and Portland^124^

*COPD* chronic obstructive pulmonary disease *IPF* idiopathic pulmonary fibrosis, *CF* cystic fibrosis, *BALF* bronchoalveolar lavage fluid, *LPS* lipopolysaccharide.

Increasing studies have also approached to the role of intratumor tissue microbiome in cancer development and therapies. T. Geller et al. found that intratumor bacteria might contribute to gemcitabine resistance of pancreatic ductal adenocarcinoma (PDAC), in which 76% of 133 human PDACs were positive for bacteria^136^. And alpha-diversity in tumor microbiome and an intra-tumoral microbiome signature were highly correlated with long-tern survival in patients. They also proved that human-into-mice fecal microbiota transplantation (FMT) experiments differentially affected tumor growth, as well as tumor immune infiltration through modulating the tumor microbiome^137^. To identify the characterization of the tumor microbiome, researchers from Israel conducted a comprehensive analysis of 1526 tumor tissues with adjacent normal tissues from seven cancer types (breast, lung, ovary, pancreas, melanoma, bone, and brain tumors) which revealed that the intratumor bacteria were mostly intracellular in both cancer and immune cells and supported significant correlations between intratumor bacteria or their predicted functions with tumor types and subtypes, patients’ smoking status, and the response to immunotherapy^96^. In addition to bacteria, a pan-cancer comprehensive analysis based on the International Cancer Genomic Consortium (ICGC) and the Cancer Genome Atlas (TCGA) has drawn a landscape of viral associations in human cancers, which detected a high prevalence of known tumor-associated viruses^138^. Yu et al. profiled the lung tissue microbiota and linked its composition and diversity to human lifestyle and clinical outcomes^93^. Also, Liu et al. reported that profile of lung cancer tissue microbiota is distinct from emphysema^89^. Consistently, Peters et al. demonstrated the existence of associations in lung tumor or normal tissue microbiome diversity and composition with patient recurrence-free (RFS) and disease-free survival (DFS)^90^. Above all, there is still an urgent need for much more explorations approaching the potentials of intra-tumor microbiome in cancer development and therapies.

**Fig. 2:**
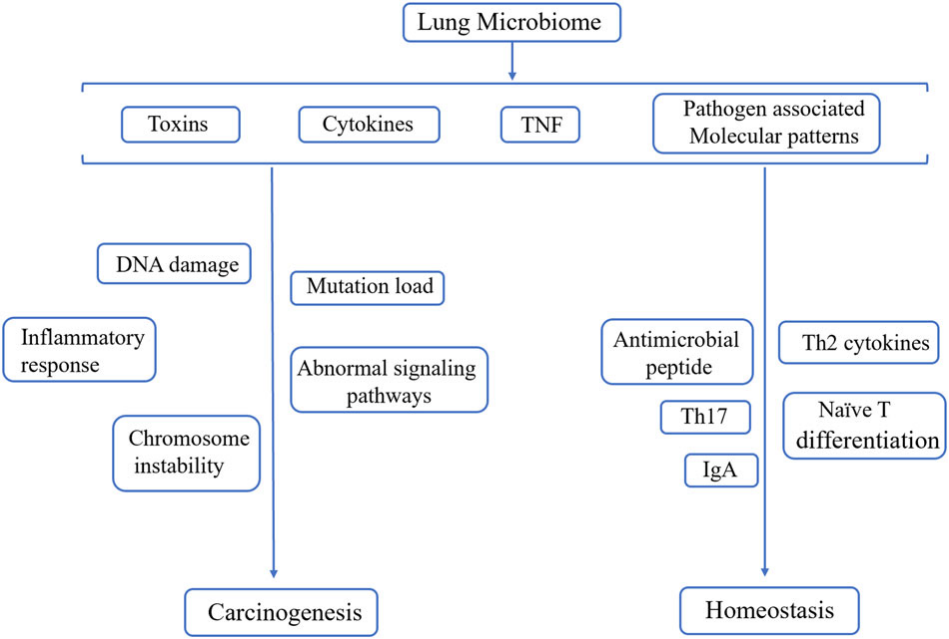
Lung microbiome play a dual role in promoting carcinogenesis and maintaining homeostasis in different conditions. Lung microbiome can induce carcinogenesis via causing DNA damage, inflammatory response alterations, chromosome instability, abnormal signaling pathway activations and increasing mutation load through productions of bacterial toxins and multiple cytokines. On the other side, the process of colonization and maturation lung-resident microbiome community also participates in the maturation of lung and promoting host homeostasis and tolerance, as well as conferring susceptibility to lung disorders during exposure to complex external environment.

Furthermore, some pioneer preclinical studies have tried to utilize GF mice (germ-free mice, free of all microorganisms including those typically found in the gut) and SPF mice (specific pathogen-free mice, free of a specific list of testing pathogens including disease-causing or research-affecting or mice health-related pathogens, opportunistic and commensal organisms in normal, healthy mice) model to elucidate the real and precise correlations between lung microbiome and lung cancer closely^82,139^. It was shown that bacteria in the lung may create a proinflammatory environment that promote lung cancer progression in the murine mouse model^82^. The GF or antibiotic treated mice were significantly protected from lung cancer development induced by Kras mutation and p53 loss when compared with SPF mice. And the commensal bacteria stimulated production of Myd88-dependent IL-1β and IL-2β from myeloid cells, inducing proliferation and activation of Vγ6^+^Vγ1^+^ γδ T cells that produced IL-17 and other effector molecules to promote inflammation and tumor cell proliferation. A similar result was also discovered by K.L. Greathouse et al. that a lower alpha diversity in normal lung as compared to non-tumor adjacent or tumor tissue and a separate group of taxa are identified, in which *Acidovorax* is enriched in smokers with squamous cell carcinoma^84^. A reasonable hypothesis is that lung microbiota homeostasis may promote the pathological processes of lung cancer. Interestingly, it was observed in spontaneous colon tumor models that the morbidity of solid tumors was significantly lower in germ-free mice compared to mice raised conventionally^140^. Furthermore, high relative microbial abundance and diversity were found to be positively correlated with patient’s better responses to PD-1-based immunotherapy treatment^141^. On the other hand, the process of colonization and maturation of lung-resident microbiome community also participates in the maturation of lung and promotes host homeostasis and tolerance, as well as confers susceptibility to lung disorders during exposure to complex external environment^35,142^. Gollwitzer et al. allergic airway inflammation was significantly attenuated in adult mice, due to an increase in expression of the surface ligands PD-L1, PD-L2, and CD40 after HDM (house dust mites) treatment^80^. Nevertheless, current knowledge cannot elaborate the causal relationships of lung microbiome alterations accompanied with disease progression, since most researches are based on long-term observations and cohort studies. It is more likely that lung microbiome may play a dual role in maintaining body stability and promoting cancer as shown in Fig. 2. However, there is no widely accepted consensus definitions for healthy or harmful lung microbiota. Moreover, there are few studies on the differences in the function of microbes in the intestine and lungs. Therefore, more large-scale clinical studies should be established to avoid errors in preclinical researches.

## Microbial effects on lung cancer treatment

Currently, the traditional treatments of lung cancer can be categorized into surgical resection, radiotherapy, chemotherapy, and immunotherapy. Even now, nearly 75% of the lung cancer patients have developed into advanced stage at the time of diagnosis (stage III/IV)^143^. Patients with advanced lung cancer always suffer from poor prognosis and limited treatment options, despite substantial advances in the oncological management of lung cancer achieved during the past three decades. Therefore, early detection and improved treatments for lung cancer are becoming increasingly urgent. The current explorations of the clinical applications of microorganisms remain at its early stages including probiotics, diet interventions and FMT (fecal microbiota transplants) in the preclinical model. Understanding the relationship between human microbiota, especially gut microbiota and lung cancer, may open a new window for the diagnosis and treatment of lung cancer.

### Microbial biomarkers

Nowadays, the widely used and effective diagnostic tools for lung cancer are chest X-ray and CT in clinic. However, the examination by low-dose spiral CT is still unable to fully popularized due to its high cost and inconvenience. The best option for lung cancer screening is to examine groups with high-risk disease characteristics including age, gender, long-term smoking, and occupational exposure. Different from the previous methods, it will be better to explore the interaction between gut microbiota and lung cancer, and try to find the microbial alterations and specific microbes that are closely associated with lung cancer, which can provide better targets to pick out the high-risk groups for chest X-ray and CT. With development and popularization of deep sequencing, the associations between microbiota in different human body sites and various diseases have held interests from both researchers and clinicians. It has been reported that there is a significant correlation between microbiota landscape alterations and development of various cancer including lung cancer^85^, melanoma^144^ and pancreatic ductal adenocarcinoma^145^. As shown in Table 1, there were many long-term observations and epidemiological studies which detected significant correlations between microbiota and lung cancer based on various sample sources^85,86,88,91,94^. Previous studies have provided novel insights into the microbiota alteration during the development and exacerbation of various lung diseases, which help establish a non-invasive detection method^106,107,110,111,118^. Zheng et al. identified and established the specific gut microbial signatures for the prediction of early-stage lung cancer^94^. Yan et al. found that *Neisseria, Steptococcus, and Porphyromonas* were significantly higher in the saliva from lung cancer patients, which may serve as potential biomarkers for the disease detection/classification^92^. A pilot study using 16S rRNA sequencing revealed that greater abundance of Bacteroidaceae, Lachnospiraceae, and Ruminococcaceae in lung tissue were significantly associated with a decreased risk of recurrence-free (RFS) and disease-free survival (DFS)^90^. There is no doubt that further clinical studies are necessary to establish microbial markers for predicting lung cancer in the future. Moreover, the precise role of salivary, sputum and feces microbiome in lung cancer initiation and progression is largely unknown and the potential molecular mechanisms awaits further investigation.

### Radiotherapy and chemotherapy

Radiotherapy for advanced lung cancer has become a routine treatment in clinical practice, although it brings some unexpected side effects such as immune damage and radiation-induced toxicity. However, the relationship between gut microbiome and ionizing radiotherapy of cancer remains unclear as there was not too much progresses in this field. A recent study revealed that mice fecal microbiota transplantation could reduce radiation-induced damage without promoting cancer cell proliferation and migration in vivo^146^. Furthermore, a unique microbial signature with enhanced IL-1β, IL-6, and TNF-α expression compared with naïve microbiota, was observed in post-radiation mouse model tissue^147^. It is promising to identify microbes hypersensitive to radiation as predictive biotargets for improvement of curative effects. Microbiota might serve as a therapeutic strategy to reduce radiation-induced toxicity and improve the prognosis of lung cancer patients after radiotherapy^148^. Recent studies have implicated that gut microbiome plays a crucial role in drug metabolism, chemotherapy-induced toxicity and host response sensitivity^149^. The gut microbiota can directly modulate drug absorption and metabolism via microorganisms and microbial enzymes^150,151^. In addition, gut microbiota can also indirectly affect the rate of metabolism in oral and systemic administration via regulating gene expression, local mucosal barrier response, and the physiology of distant organs^152,153^. Experiments in vivo and in vitro have indicated a complex and multi-level intervention relationships between chemotherapeutic agents and human microbiota. Some special microbial species and metabolites have been confirmed to inhibit the antineoplastic drug gemcitabine and promote the prodrug CB1954 in blood circulation^154^. Another subset of mainstream antineoplastic drugs is genotoxic platinum drugs, exhibiting anti-cancer effect via inhibition of DNA replication and by targeting plasma membranes and mitochondria, which leads to adverse drug reactions including intestinal toxicity, nephrotoxicity, blood–brain barrier integrity disorder and deafness^155,156^. A possible explanation is that destruction of the intestinal mucosal barrier enables microorganisms or pathogens invade into the mesenteric lymph nodes and blood circulation^157^. Furthermore, accumulating studies prove that effects of antineoplastic drugs with broad spectrum can be reduced, and antibiotics abuse may not only aggravate the side effects of anti-tumor drugs but also cause severe additional systemic side-effects^149,158,159^. At present, most studies of microbiome and chemotherapeutic drugs remains in the stage of animal experiments, and there is only little research directly exploring the gut microbiota alterations and functions in post-chemotherapy patients with lung cancer. Additional mechanistic studies and clinical trials are still required to investigate whether modulation model of gut microbiota could become an effective clinical approach to assist treatment of lung cancer with chemotherapeutic drugs and minimize drug-induced toxicity.

### Immunotherapy

It was previously reported that the disorder of the intestinal microbiota may affect the immunotherapeutic effect for cancer^160,161^. For example, the microbiome of 249 cancer patients who underwent PD-1 immunotherapy was examined by a French research group. Among them, 69 patients were given antibiotics due to other diseases at the beginning of treatment which will disrupt the intestinal flora. Surprisingly, patients treated with antibiotics have shorter cancer-recurrence time and survival time than those who did not receive antibiotics, indicating that antibiotics consumption could greatly reduce the effectiveness of immunotherapy^141^. A follow-up study compared the gut microbiota of the two groups of patients and isolated the probiotics *Akkermansia muciniphila* from the stool of the recovered patients. And this probiotic has been proved to be effective in the prevention of obesity and diabetes in previous studies. Interestingly, this study also demonstrates its contributions to cancer immunotherapy. Moreover, researchers implanted the feces of the recovered patients into germ free mice and those who received “effective” feces was responding quickly to PD-1 inhibitors. In addition, the oral probiotic *Akkermansia muciniphila* can also restore the same effect of immunotherapy^162^. One possible reason is that a higher diversity of microbiome communities might be positively correlated with T cell activity, which in turn causes cancer cells to be killed more thoroughly. Inversely, patients with “bad bacteria” have more regulatory T cells which could suppress the host immune response. A recent study of Chinese patients with advanced non-small cell lung cancer who treated with the immunological checkpoint inhibitor PD-1 showed that patients with higher gut microbiota diversity presented better response to anti-PD-1 immuno-checkpoint inhibitors. Patients with favorable gut microbiome (such as those with high diversity) exhibit enhanced memory T cell and natural killer cell signatures in the periphery blood^163^. Shi et al. revealed that systemic administration of *Bifidobacterium* potently stimulated STING signaling and increased cross-priming of dendritic cells after anti-CD47 treatment, which converts the non-responder mice into responder^164^. Paulo et al. brought forward a hypothesis that gut microbiome may help prime an immune response through TLR4-signaling^165^. Tumor-associated myeloid cells might be activated by commensal gut bacteria (via TLR4 signaling) to produce TNF and other inflammatory cytokines that mediate the tumor microenvironment and anti-tumor effect of immunotherapy^159^. After treatment with cyclophosphamide, the translocation of specific commensal bacteria into mesenteric lymph nodes enhanced Th17 responses in the spleen and the induction of memory Th1 responses, which proved the important role of commensal microorganisms in MyD88 and TLR signaling^158^. Intriguingly, a new research demonstrated that the commensal lung bacteria stimulated the production of Myd88-dependent IL-1β, IL-2β, and IL-17 and other effector molecules to promote inflammation and tumor cell proliferation^82^. These studies revealed a close relationship between intestinal flora and cancer immunotherapy which provided new insights for improving the effectiveness of tumor immunotherapy. Nevertheless, further understanding of the effects and mechanisms of microbiome and immunotherapy needs more explorations.

### Probiotics, prebiotics, and microbial targeting drug

At present, mature products targeting microbiome that have entered the commercial market include probiotics, prebiotics, and synbiotics, having showed generally safety in different clinical practice. The general effects revealed by increasing clinical data include promoting gastrointestinal homeostasis and integrity, regulating metabolism via productions of SCFAs (short-chain fatty acids) and vitamin or second bile salts, participating digestive activities and neutralization of inflammation and carcinogens^166^. One of the major effects of administration of prebiotics and probiotics is to achieve optimal host immune homeostasis through maintaining the diversity and relative numbers of *Bacteroidetes*, *Firmicutes*, *Proteobacteria*, *Actinobacteria* and so on^167,168^. On the other hand, the pharmaceutical and biotechnology companies worldwide are looking for microbial drug targets for developing and promoting chemotherapy and targeted therapy for cancer. Drug targeting microbiome have potentials to lessen the side effects resulting from chemotherapy, in which a clinical trial suggested a clinical benefit from administering neomycin concurrent with irinotecan to diminish the side effects^169^. In another preclinical study, mice treated with the novel small-molecule inhibitors targeting bacterial β-glucuronidase were protected from irinotecan (an anticancer drug) induced diarrhea^170,171^. However, the current limited investigation and knowledge about the beneficial microbiota and molecular mechanisms cannot provide the best method to dissect the host microbiome. Whether the microbial changes will cause unexpected local homeostasis disorders, inflammatory response or even precancerous lesions remains elusive. Recently, FDA issued a safety alert on the application of FMT for the risk of serious adverse events due to transmission of pathogenic organisms^172^. Even though emerging achievements have indicated a promising application of microbiome in anti-cancer action, future studies should focus more on the causal effects of lung microbiome alterations on lung diseases and identify the healthy and beneficial lung microbiome.

## Discussion and perspectives

**Fig. 3:**
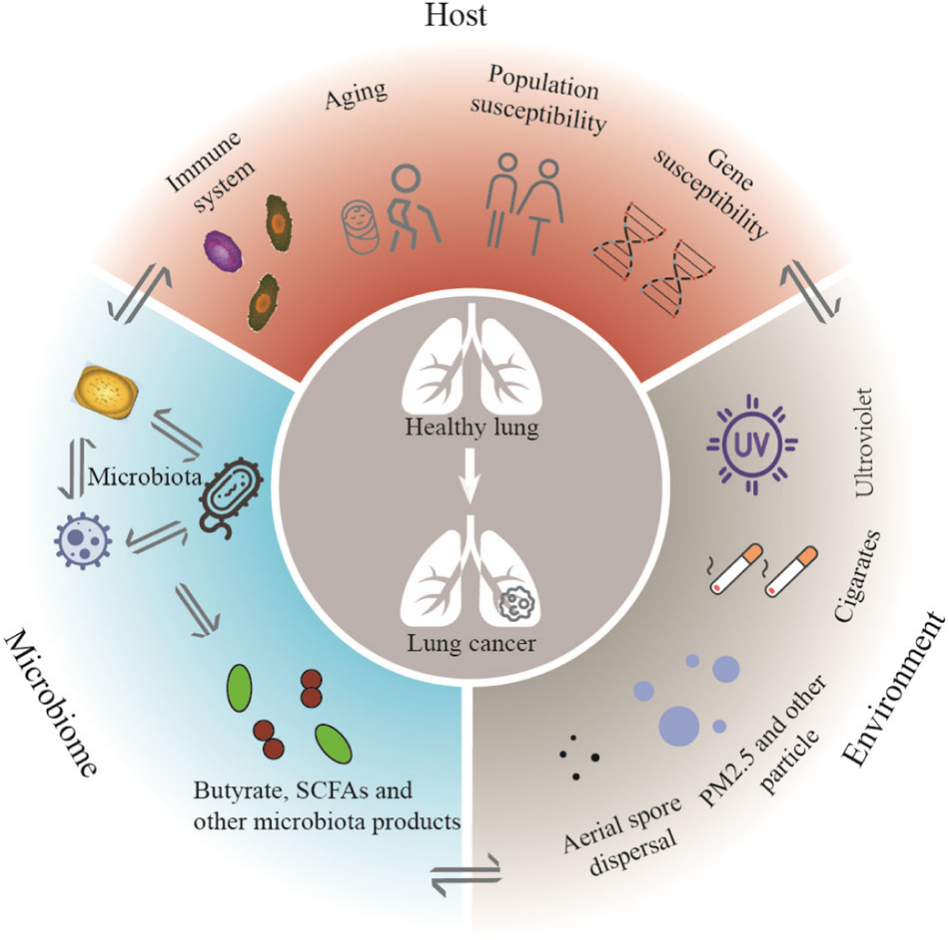
“One Health” care of lung: triple interaction among host, microbiome, and environment during lung cancer progression. Aging, population or gene susceptibility are associated with tumorigenesis. As extrinsic factors, the microbiota produced the cytotoxicity-related components, inducing the DNA damage of host cells. The microbiota and its metabolites (e.g., short-chain fatty acids (SCFAs)) trigger downstream immune and metabolic signaling pathways, which further promote or suppress the malignant behaviors of host cells. Environment factors (ultraviolet ray, cigarette and particles) can cause altered community of microbiota and gene mutation to promote occurrence of lung cancer.

Lung cancer has been the leading cause of cancer-related deaths worldwide mainly due to its initially asymptomatic and typically diagnosis at an advanced stage^3^. As shown in Fig. 3, the triple interaction among host, microbiome and environment maintains lung homeostasis in healthy functioning. Moreover, the microbiome potentially possesses inestimable therapeutic strategies in promoting conventional lung cancer treatments including radiotherapy, chemotherapy, surgical resection, and immunotherapy. Undoubtedly, microbiome was confirmed to be involved in various diseases initiation and development. But the complex mechanisms remain unknown. Collectively, there are still some critical problems in this field: First, there are many studies investigating the role of gut microbiome in various lung diseases^63,94,173–175^. It will be very interesting to dissect the lung-resident microbiome but there are technical challenges regarding to the characterization of the low-biomass lung microbiome by the next generation sequencing technologies^176,177^. Second, the role of microbial components such as fungi and virus other than bacteria, is largely unexplored in lung cancer, partly due to their relatively lower abundance and lack of well-characterized reference genomes. Third, currently most studies are conducted in laboratory mice. The difference in microbiome structure between human and mice can result in limited translational potential from murine studies to human diseases. Therefore, more effective and human relevant preclinical models approaching the causal relationship between microbiome and tumor is particularly important. For example, the development of patient derived organoid will be a good option^178–180^. Fourth, the role of microbiota in the progression of lung cancer has attracted more attention to the interaction between tumor immune microenvironment and microbiota^82^, but lung tumor microenvironment derived microbiota was also reported to acted directly on the tumor cells^84,91^. Finally, there’s an associated effect between the gut microbiota and immunotherapy in the advanced NSCLC^141,163^. And the microbial biomarkers in early-stage lung cancer, or probiotics for treatment of lung cancer still need to be explored. Although the huge potential of microbiome has drawn a great landscape for prevention and treatment of lung cancer, it was generally recognized that development of this field requires more multidisciplinary and in-depth exploration. A better understanding of microbiome in the process of cancer initiation and different response to multiple treatments may provide great opportunities for promoting diagnosis and prognosis of lung cancers patients.
